# Cost Savings with Rapid Diagnostic Tests for Malaria in Low-Transmission Areas: Evidence from Dar es Salaam, Tanzania

**DOI:** 10.4269/ajtmh.2010.09-0632

**Published:** 2010-07

**Authors:** Joshua Yukich, Valerie D'Acremont, Judith Kahama, Ndeniria Swai, Christian Lengeler

**Affiliations:** Department of International Health and Development, Tulane University School of Public Health and Tropical Medicine, New Orleans, Louisiana; Swiss Tropical and Public Health Institute, Basel, Switzerland; University of Basel, Basel, Switzerland; City Medical Office of Health, Dar Es Salaam City Council, Dar Es Salaam, Tanzania

## Abstract

Rapid diagnostic tests (RDTs) for malaria may help rationalize antimalarial drug use. However, the economic effects of these tests may vary. Data on costs were collected from 259 patients in 6 health facilities by using exit and in-charge interviews and record reviews during a trial of RDT rollout in Dar es Salaam, Tanzania. The RDTs decreased patient expenditure on drugs (savings = U.S. $0.36; *P* = 0.002) and provider drug costs (savings = U.S. $0.43; *P* = 0.034) compared with control facilities. However, RDT introduction did not significantly reduce patients' overall expenditures (U.S. $1.02, 95% confidence interval [CI] = $0.76–$1.36 versus U.S. $1.33 95% CI = $0.99–$1.77) and may increase total provider costs (U.S. $3.63, 95% CI = $3.40–$3.89 versus U.S. $2.32, 95% CI = $1.99–$2.69) compared with control facilities. Clinician's compliance with test results was higher with RDTs than with routine microscopy (95% versus 82%; *P* = 0.002). The RDTs reduced drug costs in this setting but did not offset the cost of the tests, although they also resulted in non-monetary benefits, including improved management of patients and increased compliance with test results.

## Introduction

Within public health facilities in Africa, malaria is largely diagnosed on clinical grounds alone, and fever cases are routinely treated without laboratory confirmation.^1^ Malaria microscopy, when available, is often of poor quality.^2^,^3^ However, with the high costs of the new-generation artemisinin combination therapy (ACT), and concerns about development of drug resistance caused by drug overuse, many donors and health system managers are searching for ways to improve the rational use of drugs for malaria treatment. Additionally, in many malaria-endemic areas, intense malaria control activities and rapid urbanization have led to decreasing clinical malaria incidence rates. As a consequence, the malaria-attributable rates in fever episodes have been decreasing, further increasing the need for improved diagnostic strategies.^4^

The modern generation of histidine-rich protein 2 (HRP2) antigen-based rapid diagnostic tests (RDTs) has been shown in trials to have high sensitivity and specificity for the diagnosis of *Plasmodium falciparum* infection among clinical patients in Africa.^5^,^6^ Several studies have shown that the sensitivity of RDTs can be higher than expert microscopy and thus far more accurate than routine microscopy.^6^–^9^

There are potential drawbacks to the use of RDTs for routine malaria diagnosis, including the persistence of the target antigen in the bloodstream for up to several weeks after an infection has been treated. Thus, RDTs cannot be used to measure treatment success.^10^–^12^ Additionally, such tests cannot determine parasite load and their specificity may vary with the setting.^5^

Although more accurate laboratory diagnosis may help to rationalize anti-malarial drug use at health facilities in Africa, there is also a clear possibility that any cost savings made from the reduction of anti-malarial prescriptions may be outweighed by increases in prescription of antibiotics or other drugs to treat patients with negative test results.^13^ Additionally, RDTs add cost to case management, which is not inversely proportional to the number of patients tested, as is the case for microscopy, and which could outweigh cost savings from reduced anti-malarial consumption.^14^ The effects of diagnostic changes will also depend on the adherence of clinicians to the diagnostic result, the frequency with which they request a test, and the prevalence of parasitemia among the clinical population.^15^,^16^

There is a sizable body of literature in which the implications of improving malaria diagnostic methods, including empirical and modeling studies, have been reported. The results of the empirical studies have shown it may be possible to reduce average cost per patient and household costs through improved malaria diagnosis and that such interventions could be implemented in a highly cost-effective manner.^13^,^16^–^18^

Modeling studies have helped to confirm and highlight the myriad factors that could influence the cost-effectiveness and overall cost-saving potential of improved diagnosis. The main factors that influence the desirability of one testing strategy over another relate to the proportion of febrile cases that are parasite positive, the sensitivity and specificity of the new method and its alternatives, the costs of the tests, clinicians' adherence to test results, and the cost of drug regimens prescribed to parasite-positive and parasite-negative patients.^13^,^14^,^16^–^20^

Because the potential for cost savings appears to be highly situation dependant, it is necessary to evaluate the economic implications of the decision to shift to RDTs locally and at specific levels of the health care system. Although models can be used to explore these implications, there is still a strong rationale to assess alternatives and validate models empirically in representative settings.^21^ This report describes a study of the economic implications of the implementation of diagnosis with RDTs in a low-endemicity urban African setting in Dar es Salaam, Tanzania.

## Materials and Methods

### Study area.

Dar es Salaam is the economic capital of Tanzania. It is a large urban area (population approximately three million) with highly heterogeneous land use, including commercial districts, industrial districts, residential districts, urban slums, and areas with high levels of urban agriculture.^22^ As a result, there is high variability in the number of *Anopheles* spp. breeding sites and thus adult mosquito densities across the city. As of 2009, this city is considered a low but stable malaria transmission area: entomologic inoculation rate »1.3, with low parasite prevalence (< 10% in the general population).^23^ All health facilities included in the costing exercise are located in densely populated low-income areas, although the catchment area of one facility (Kawe Dispensary) also includes some peri-urban and higher-income areas.

Twelve public health facilities (three hospitals and nine primary health care facilities) were selected for inclusion in a trial of RDT rollout. The three hospitals were assigned to receive the RDT intervention, and the nine primary care facilities were randomly assigned to either receive the RDT intervention (experimental) or not receive it (control).^24^ Of these facilities, six primary health care facilities were included in the costing exercise. Four primary care facilities were experimental facilities in which RDTs replaced routine microscopy for the diagnosis of malaria. Two primary care health facilities remained as controls with only routine microscopy. These six facilities were selected because this evaluation was targeted at the primary care level, and these facilities were the most comparable in terms of patient population, size, and numbers of monthly consultations. Sample size was calculated using EpiInfo version 3.4.1 (StatCalc Module; Centers for Disease Control and Prevention, Atlanta, GA) with the aim of being able to measure a 25% difference in arithmetic mean patient costs between RDT and control facilities with 95% significance and 80% power.

### Collection of patient and facility costs and resource use.

Costing was conducted from the patient and provider perspectives. A large survey examining the effects of RDT introduction on health worker practices, and patient response was used as a platform for collecting patient-specific and facility costs. This survey was the second in a pre–post cross-sectional survey evaluation of the RDT intervention (that consisted of training of health workers in February 2007, initiation of RDT use at the end of March 2007 and supervision on site 1, 2, 5, 10, and 15 months after RDT introduction) and conducted 15–18 months post-intervention from July through September 2008. Within the six selected facilities, inclusion criteria were 1) first consultation for the present complaint (not a follow-up visit), 2) absence of severe disease, and 3) main complain not trauma related. Eligible patients or caretakers of young patients were included if they gave oral informed consent to participate. Their consultation was then passively observed by a survey worker with clinical training. These patients or caretakers were questioned during an exit interview, which included questions relating to their perceptions of the clinical and laboratory consultation, and episode-related expenditures and any previous treatment seeking and related expenditures. The questionnaire also probed travel time and costs, time spent accessing the facility, and missed work or lost income due to attendance at the facility and/or any time taken to care for the patient at home.

All patients or caretakers who participated in the exit interview (and therefore had costing questions) were requested to return to the same health facility one week later for a follow-up interview. They were also provided with a small incentive to cover transportation costs. At follow-up, all patients or caretakers were administered a second questionnaire that solicited information on their current health status and any treatment-seeking activity or expenditure during the intervening week, and lost income and time taken to care for the patient at home. They were also asked about the previous consultation and any associated expenditures, mainly as a check on consistency, and to potentially garner information about informal payments. If in-person follow-up was not possible, we attempted a shortened follow-up interview by mobile telephone. All follow-up patients who reported at a health facility were tested with an HRP2-based RDT to check for missed infections, and to identify persons with false-negative or false-positive results in the control facilities because of the persistence of the HRP2 antigen in treated patients. Persistence of the antigen was low (< 33%) in previously RDT-positive and appropriately treated patients. Thus, such results for the second RDT were not of use for determining numbers of correct diagnoses. For the patients who were positive by RDT during follow-up, it was ascertained whether they had received appropriate first-line treatment and whether their condition had improved. If this was not the case, we ensured that they were subsequently treated for malaria at the health facility.

To assess treatment costs to the provider a health facility survey was conducted in the six facilities participating in the cost study to identify per patient resource use at the facility level. In-charge interviews and a health facility level data survey instrument were used to collect information on the number of outpatients and malaria cases seen at the facility during the past three years and on the number of blood slides and RDTs performed. Additionally, we collected information on numbers and grades of staff and estimated effort dedicated to outpatients. We also collected additional information used to calculate overhead and patient visit costs, including facility's spending on electricity, water, other overhead costs, and the numbers of capital items in the facility, including microscopes and other clinical equipment. Facility records were also used to collect information on the use of consumables including laboratory books, Giemsa stain, blood slides, lancets, syringes, and other consumables. We measured resource use at each of the six facilities through a questionnaire administered to the in-charge of health facility and by collection of routine data on facility use, numbers of outpatients treated, numbers of malaria test performed, and records of consumables used. Additionally, we conducted data collection at the central offices of the City Medical Office of Health to estimate the costs of construction of health facilities and other costs, which we could not obtain directly from the health facilities themselves, including salary ranges for various grades of health workers.

### Valuation of resource use.

Costs of resource inputs were determined for the provider costs on the basis of 1) the Tanzania pharmaceutical and supply price list for 2008, and 2) interviews with the appropriate financial managers of the Dar es Salaam City Medical Office of Health. Information on drug prices was obtained from the International Drug Price Indicator Guide database published by Management Sciences for Health or from a World Health Organization–AFRO database of indicator drug prices.^25^,^26^ Patient costs were valued according to patients reported expenditures and lost income.

Costs for the initial implementation of RDTs, including training and quarterly supervisory visits, were calculated based on reported expenditure and activities, excluding specific research costs. Costs were reported in the local currency (Tanzanian shillings [TSH]), U.S. Dollars (USD), or Swiss Francs. All costs were converted to USD by using official exchange rates for the year in which the cost occurred and adjusted into a common year (2008) using the U.S. gross domestic product deflator.^27^ Capital costs were discounted and annualized by using a 3% discount rate and assumed lifetimes for equipment based on expert opinion and past literature. All costs attributable to RDT implementation were then divided by the estimated total number of RDTs performed in the nine experimental (from the full trial) health facilities (328,000–435,000) over the entire duration of the project (approximately two years) to calculate an average implementation cost per test. The number of tests was estimated in two ways: the first and lower number was the number of RDTs performed according to facility records, and the second and larger number referred to the number of RDTs delivered to the facilities according to project records. This range had little effect on the cost of implementation per test (excluding the cost of the test itself).

For provider costs, all costs were related to their allocation to outpatient services (as opposed to maternal and child health services) and then related to the number of out-patients seen at the facility during the period when their consumption could be measured (2005–2008). Because some resource use could not be measured at each facility because of missing records (26% of requested records were missing), these costs have been estimated by using the mean values per patient from the facilities in which information could be collected. Prices of drugs have been adjusted to account for transport costs and wastage according to the following assumptions. Drugs costs were inflated 20% over actual costs to adjust for wastage, then an additional 10% for local transport, and finally an additional 10% for international transport when cost, insurance, and freight prices were not available.

### Statistical analysis.

We tested the differences in patient expenditure and provider costs by using two statistical tests. We first applied the Kruskal-Wallis test for equality of populations. We used this test because, as is typical of expenditure data, the distribution of patient-specific provider costs and patient expenditures was highly non-normal due to a significant right skew and a large zero mass. Thus, performing significance testing with a non-parametric method was necessary.

Additionally, we used non-parametric bootstrapping to estimate confidence intervals for each expenditure value. This approach was adopted because alternative non-parametric methods do not compare arithmetic mean costs, transformation of the data to a log scale would result in comparison of geometric means, and such transformation did not result in a normal expenditure distribution. Method studies and reviews have suggested this method to appropriately deal with the need to compare arithmetic means and generate confidence intervals for such data.^28^,^29^

Total time for patient visits to the health facility was normally distributed and thus amenable to standard parametric tests. Data was entered in EpiInfo 3.4.1 (Centers for Disease Control and Prevention) and analyzed by using STATA version 9.2 (Stata Corp., College Station, TX).

The study protocol and related documents were reviewed and approved by the National Institute for Medical Research Review Board in Tanzania.

## Results

### General results.

**Figure 1.**
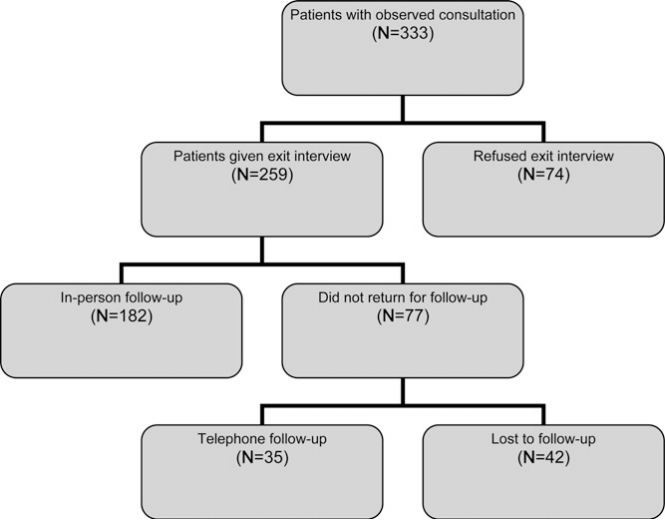
Losses to follow-up during the study, Tanzania.

A total of 333 patients were recruited during consultation at each of the six selected facilities for exit interviews, and 259 patients were successfully administered an exit interview. The final rate of follow-up (for patients participating in the full costing study) was 84% and is shown in Figure 1. No patients or clinicians refused the initial consultation observation. At the 5% significance level, patients in RDT facilities were no more likely to attend the exit interview than those in control facilities; after stratifying by the portion of the patient population more than five years of age, differences were less significant. Patients who were lost to follow-up were not significantly different from those who were successfully re-interviewed based on several demographic measures (Tables 1 and 2). No significant differences were found for age distribution, patient sex, method of travel to and from the health facilities, or occupation of the patients' head of household. Additionally, we found no significant differences for the same set of measures between facilities that offered RDTs and those facilities that did not offer RDTs (Tables 1 and 2). Unfortunately, patients who were more than five years of age were significantly more likely (29% versus 15%) to leave the health facility before completing the exit interview.

Of the 259 patients who were administered exit interviews, 178 were interviewed at experimental facilities (with RDTs) and 81 were patients at control facilities (no RDTs). Within the RDT facilities, patients were significantly less likely than in control facilities to receive results for a laboratory test for malaria (84% versus 95%; *P* = 0.009, by Fischer's exact test), a difference that was also significant in patients ≥ 5 years of age (86% in RDT facilities versus 98% in control facilities; *P* = 0.04) but not in children less than five years of age (82% in RDT facilities versus 92% in control facilities; *P* = 0.13). Patients in RDT facilities were also significantly less likely to have a positive test result for malaria parasites (14% versus 43%; *P* < 0.001). Although in control and RDT facilities large fractions of all patients received laboratory diagnosis, clinicians in the RDT facilities appeared to be more parsimonious in their use of tests, at least among adults.

Adults were significantly more likely than children less than five years of age to have positive results for malaria in control facilities, but not in RDT facilities (55% versus 28% in control facilities; *P* = 0.016 and 15% versus 13% in RDT facilities; *P* = 0.69). These results clearly confirm the problem with microscopic examination, and the low quality of routine microscopy was confirmed in more detailed studies in the same facilities.^3^

Patients within RDT facilities were significantly less likely to receive the first-line anti-malarial drug artemether/lumefantrine (ALU) (Coartem™; Novartis, Basel, Switzerland) (ALU) compared with patients in control facilities. This finding was seen when the analysis was restricted to patients who left the facility with any drug prescription and all patients observed: 12% versus 52%; *P* < 0.001 (with any prescription) and 10% versus 48%; *P* < 0.001 (all patients). This difference remained highly significant regardless of the age of the patient.

Patients in RDT facilities were also more likely to receive ALU in correct correspondence with the results of their diagnosis. When only patients with a laboratory diagnosis were examined, those in RDT clinics received ALU in correspondence with the laboratory diagnosis 95% of the time versus 82% in control facilities (*P* = 0.002). Because a related study has shown that most positive blood slide results in control facilities are false-positive results (Kahama J and others, unpublished data), it follows that high clinician compliance with microscopy results leads to overuse of anti-malarial drugs. Patients less than 5 years of age appeared no more likely to be correctly prescribed ALU than patients ≥ 5 five years of age (93% for patients < 5 years of age versus 88% for patients ≥ 5 years of age; *P* = 0.17). Differences remained statistically insignificant when restricted within either RDT facilities or within control facilities.

### Implementation costs of RDT program (provider).

Cost data on implementation was collected over a 14-month period. During this period, approximately 435,400 RDTs were issued to implementing facilities, and use data indicated that approximately 330,000 RDTs for malaria had been performed. Because of this high volume of tests, the cost of implementation training and support for RDT rollout was relatively low when considered per test. The total cost of the RDT intervention over this period (not including the test kits) was estimated to be $16,946 in 2008 USD or $1,883 USD per implementing facility. Thus, we estimated that the cost of implementation per RDT (excluding the test kits themselves) was between 0.04 USD and 0.05 USD. The test kits themselves were estimated to cost USD 0.66 each. When calculating the cost per patient in RDT clinics, we include the cost of RDT implementation.

The bulk of the expenses went to staff salaries for the implementation of the RDT rollout (72%) and for training and quality control at the implementing facilities (22%). The only other substantial line item cost was transport, which accounted for 3% of the total cost of implementation.

### Patient perspective: Direct costs (expenditure).

Patient costs consist of two main parts: direct costs due to expenditure on medicines, transport, diagnostics, or other health services, and indirect costs, such as lost productivity or the opportunity cost due to time spent seeking care. We attempted to measure direct costs and indirect costs.

Patient expenditures were directly reported by patients. Table 3 shows arithmetic mean expenditure per patient in RDT or control facilities arising before and during the first consultation, and after the first consultation for the subset of patients with follow-up. Expenditures have been subdivided into several categories, and are reported in TSH and USD.

Table 3 shows that significant differences in reported expenditure were found between patients at RDT clinics and those at control clinics. Patients' mean total expenditures were lower in RDT clinics (USD 1.02) compared with control clinics (USD 1.33), and were significantly different by the Kruskal-Wallis test for equality of populations. Patients' mean expenditure on drugs was 0.36 USD lower in RDT clinics than in control clinics.

Table 4 shows bootstrapped means and bias corrected confidence intervals for each of the parameters shown in Table 3. Each estimate is based on 10,000 re-samples of the observed data.

Arithmetic mean patient expenditures, when reduced into smaller component parts, failed to show significant differences in all but the line item expenditure for drugs at the first health facility visit, which was highly significantly different in RDT clinics (TSH = 464 [USD = 0.38] versus TSH = 902 [USD = 0.74]; *P* = 0.002, by Kruskal-Wallis test). However, bootstrapped confidence intervals showed that the difference was only close to statistical significance.

The similarity of expenditure across the two types of facilities helped to support our assumption that the populations of patients in control and RDT facilities were similar because the cost of transportation and actions taken before attending the health facility would not be expected to be significantly different between the two groups. Furthermore, it supports the argument that effects on patient expenditures were largely limited to those on drug purchases. Expenditures on drugs at the health facility accounted for the largest single component of patient expenditure, followed by laboratory fees and travel costs.

### Patient perspective: Indirect costs.

Additionally, patients incurred indirect costs through lost income, reduced productivity, and the opportunity cost of lost time caused by attending the facility either as patients or as caretakers of patients. One hundred eight (42%) patients or caretakers reported missing work to attend the health facility. Of that group, 85% reported lost income as a result. Neither result was significantly different at the 10% level between RDT and control facilities (*P* = 0.16, degrees of freedom = 1 and *P* = 0.66, degrees of freedom = 1). Among those reporting lost income, mean lost income was reported as 7,175 TSH (5.87 USD), a figure that was not significantly different between control and RDT groups (*P* = 0.16, by Kriskal-Wallis test). This figure is significantly larger than patients' expenditures on all other categories. For patients who lose income to attend the facility, the opportunity cost of facility attendance is far larger than the direct costs of health care and such large opportunity costs might prevent significant numbers of persons from accessing care.

Total time per visit, including transportation time, was measured by adding estimates of time at which patients or caretakers left their home or work place to attend the facility to the time they spent at the health facility (determined by the time of the start of their exit interview), with an additional time factor added for their estimated time to return home. In control clinics, mean time per visit was estimated to be 4.7 hours, and in RDT clinics, it was estimated to be only 4.0 hours (*t* = 2.8703, *P* = 0.005). Thus, being a patient in an RDT clinic in our sample was associated with approximately 42 minutes shorter total visit time. Although a certain amount of this variation can be attributed to slightly shorter travel times to RDT facilities (mean travel time = 35 minutes) compared with control facilities (44 minutes; *P* = 0.058), we observed a mean difference of approximately 9 minutes of travel in each direction, or a total of 18 minutes. Reduced waiting times and total visit times might help to reduce the opportunity costs of facility attendance and thus could improve access to care, although reductions seen are small (< 10%) in relation to total visit time.

### Provider perspective.

In this analysis, we focus on gross provider economic costs and not net costs, which would account for the collection of user fees by health facilities. Table 5 lists the costs that were included in the analysis.

Table 6 shows the results of the provider perspective analysis for the RDT and control facilities. The table shows the results of non-parametric tests for each of three sub-divisions of total provider costs. These costs are analyzed either within control or experimental facilities. Drug costs represent the cost to the provider of all drugs and prescription provided to a given patient. Facility cost is the cost of the commodities whose use is measured at the facility level but not linked to specific patients (overhead, staff costs, equipment, and general consumables, excluding drug costs). Thus, there are only six observations corresponding to the number of facilities in our study. Total costs include drug costs, facility costs, and other marginal costs, including RDTs and consumables, which are patient specific rather than general consumables used for all patients. Significant differences were found for all costs except facility cost, although the latter result is compromised by the extremely small sample size.

When patients who attended RDT clinics were compared with patients who attended control clinics, drug costs were significantly lower for patients who attended RDT clinics (USD 1.28 versus USD 1.71; *P* = 0.014). However, total provider costs were higher for patients who attended RDT clinics (USD 3.63 versus USD 2.32; *P* < 0.001 for total cost).

We were again confronted with results that were highly non-normally distributed, including in some cases a large zero mass and in all cases a significantly right-skewed distribution. Thus, we estimated confidence intervals by using non-parametric re-sampling (bootstrapping) with 10,000 re-samples for each outcome, excepting facility cost (Table 7).

Bootstrapped confidence intervals generally confirm the results of the Kruskal-Wallis tests. However, there are important differences between the two results. The confidence intervals for drug costs between RDT and control facilities show a large overlap when analyzed for all age groups. However, when the sample is stratified by those < 5 years of age and those ≥ 5 years of age, a significant difference exists for patients ≥ 5 years of age. This finding may be the result of a combination of high rates of ALU prescriptions in control facilities and the higher cost of this drug for adults in relation to other adult drugs. Once facility costs are included, the total cost of treating a patient, including all provider costs, is significantly higher in RDT facilities than in control facilities.

### Summary of results.

The results indicate that in the presence of RDTs, drug cost savings are likely to accrue to patients, and may also accrue to the providers, especially for adults. However, whether these savings translate into overall cost savings is unclear. For patients, it is likely that there is some reduced overall spending when RDTs are available. However, the savings is small (USD 0.36) and it represents only a small component of the total economic costs to patients.

For providers, the drug cost savings is of a similar order (USD 0.43) as a result of RDT introduction. Unfortunately, these savings appear to be too small to offset the entire cost of RDT introduction and use. Thus, it appears that RDTs may increase the cost of treatment per patient in public facilities, despite reducing anti-malarial drug use and creating drug cost savings for the health system. Additionally, the cost savings arise largely from reduced anti-malarial use among adults who are most likely to be charged a user fee for drugs. Thus, the resulting reduction in user fee revenue caused by reduced patient drug expenditure will reduce the financial incentives for RDT implementation.

## Discussion

This study was conducted under routine conditions in health facilities and sampled patients were taken from six health facilities. Because there is likely to be a tendency towards similar prescribing practices within facilities, the results should be adjusted for clustering within health facilities.^30^ Unfortunately, because of the small sample size and small number of health facilities included in the study, we were unable to formally account for this in most of the statistical analysis. Nevertheless, we believe that these results are likely to be robust, although the extent to which they are generalizable depends on how representative these facilities are of typical facilities in Tanzania or more widely of other health systems in sub-Saharan Africa.

These results are sensitive to the relative prices of malaria drugs and antibiotics (ALU: USD 0.41 to USD 1.60 depending on dosage and antibiotics: USD 0.20 to USD 3.50 depending on drug, dosage, and formulation) and any other drugs used to treat patients with positive and negative test results. It is possible that given changes in drug prices, our results could change. One of the most expensive drugs commonly used among these patients is ACT for malaria. Much of the drug cost savings seen in this study to patients and providers is caused by reduced anti-malarial use in RDT facilities. Thus, a reduction in the price of anti-malarial drugs may eliminate the drug cost savings we observed. Currently, a global subsidy scheme for antimalarial drugs is at an advanced stage and a virtually free drug at the country level might be a disincentive to testing for malaria.^31^ Subsidized provision of malaria RDTs should be considered in parallel to help overcome this problem.

Additionally, we chose to focus on gross provider costs. This focus could affect our results because it excludes health facility receipts for user fees. However, based on the evidence from the perspective of patients, the primary effect of RDTs was likely to have been a reduction in user fees caused by reduced prescriptions of drugs and no changes in laboratory or general fees. Thus, shifting the perspective to net costs at the facility level would have likely made RDTs appear even more costly than microscopy because they reduce the primary source of health facility user fee revenue.

It has been postulated in several studies that RDTs could produce cost savings to health facilities in low transmission settings.^18^,^19^ We show based on empirical data from six health facilities in Dar Es Salaam that significant drug cost savings and reductions in anti-malarial drug use appear to be achievable in such settings. In addition, there was a large reduction in false-positive malaria test results because of good specificity of routine RDTs. Clinicians' compliance with test results was also significantly better when RDTs were in use, and this is likely to have contributed to the drug cost savings seen we observed.^16^ Increased compliance by providers may be a result of high-quality training or their perception of the increased accuracy of RDTs compared with their routine laboratory diagnostic methods.

However, despite demonstrable drug cost savings, we did not see overall cost savings caused by use of RDTs. Provider costs appeared to significantly increase in the presence of RDTs. Although some of this increase is likely caused by higher fixed costs within the set of facilities selected for this study, the drug cost savings that we observed (USD 0.43) were not likely to be large enough to offset the cost of the RDTs when a high percentage (84%) of the patients at the facility have the test administered. Furthermore, reduced patient expenditure on drugs leads to decreasing revenue from user fees, which might make the intervention appear economically less attractive at the facility level. Other considerations such as reduced effort at the laboratory level may partially compensate this loss.

Our results show that for a significant proportion of all persons seeking treatment, the indirect costs of lost productivity far outweigh the direct costs of transportation and treatment. When averaged over all the patients observed, lost income accounted for approximately two-thirds of all costs. Other studies have had mixed conclusions about the balance of direct and indirect costs of uncomplicated malaria morbidity. However, there is a general consensus that indirect costs of morbidity form an important part of the economic burden of malaria.^32^ Our results are consistent with a number of studies from Africa and other locations, which showed that indirect costs of malaria can outweigh direct costs of treatment for uncomplicated episodes.^33^–^36^

Unfortunately, in the context of this study, it was not possible to measure differences in health outcomes between the groups of patients, or to assess whether patients were truly malaria infected by using expert microscopy. Thus, it was not possible to assess the cost-effectiveness of the intervention, either per health outcome or per additional correct diagnosis. An in-depth analysis of case management practices will determine if RDTs improved compliance with standard treatment guidelines and improved case management (d'Acremont V and others, unpublished data). That information could also be used to calculate a cost per additional correct treatment, as has been done in previous diagnostic costing studies.^14^

An additional benefit is that with a more reliable laboratory test, it becomes more realistic to monitor malaria trends on the basis of routine data. Although it is unclear what monetary value such a benefit would have for the health system, substantially improved knowledge of malaria incidence rates could lead to efficiencies in other health care domains. Distribution of malaria prevention and treatment resources could potentially be more efficiently allocated to high-incidence areas. Alternatively, more accurate measures of malaria incidence could enhance responsiveness of health systems to malaria epidemics.^37^ Finally, reduction of unnecessary anti-malarial over-use is important to limit the development of resistance to ACTs.^38^

Our results show significant savings on drug expenditure to patients and on drug costs to providers in the presence of RDTs. However, the savings are outweighed by other fees and charges and lost income for patients, or the cost for RDTs and higher facility costs for providers.

Clinicians' compliance with test results was higher when RDTs were used, which showed that they trusted this new technology. It is also likely that use of such tests accrues significant other benefits, including improved case management, more rational anti-malarial drug use, and reductions in development of resistance to ACTs. Although valuation of such benefits is outside of the scope of this work, they are highly important from a public health perspective. Although RDTs are likely to bring significant benefits to the health system in areas such as Dar es Salaam, these benefits may not be fully paid for through drug cost savings, but require additional investment.

## Figures and Tables

**Table 1:** Comparability of control and experimental populations, and those lost to follow-up, Tanzania*

Characteristic	No.	Estimate	95% Confidence interval	*P*†
Proportion of patients > 5 years of age				
Control facility	81	51.9	41.0–62.7	0.49
RDT facility	178	47.2	39.9–54.5	
Lost to follow-up	42	59.5	44.7–74.4	0.12
Not lost to follow-up	217	46.5	39.9–53.2	
Proportion of patients who were female				
Control facility	81	55.6	44.7–66.4	0.87
RDT facility	178	54.5	47.2–61.8	
Lost to follow-up	42	54.8	39.7–69.8	0.99
Not lost to follow-up	217	54.8	48.2−61.5	

* RDT = rapid diagnostic test.

† By Pearson's chi-square test (degrees of freedom = 1).

**Table 2:** Comparability of control and experimental populations, and those lost to follow-up, Tanzania*

Patient method of travel to health facility	Lost to follow-up, n = 42	Not lost to follow-up, n = 214	RDT facility, n = 175	Control facility, n = 81
Walking	61.7	60.8	61.7	61.7
Mini-bus	33.3	35.5	34.9	35.8
Other	0.0	3.7	3.4	2.5
*P*†	0.529		1.000	

* RDT = rapid diagnostic test.

† By Fischer's exact test.

**Table 3:** Patient expenditures, Tanzania*

Type of expenditure	Facility	No.	Mean cost per patient		Standard deviation (TSH)	*P*†
			TSH	U.S. $		
Care pre-HF	RDT	178	89	0.07	630	0.506
	Control	81	46	0.04	298	
Drugs at HF	RDT	178	464	0.38	1,060	0.002
	Control	81	902	0.74	1,407	
Out-patient charges	RDT	125	79	0.06	117	0.347
	Control	56	104	0.08	129	
Laboratory fee at HF	RDT	122	245	0.20	411	0.841
	Control	56	252	0.21	431	
Post visit	RDT	126	198	0.16	1,008	0.956
	Control	56	70	0.06	447	
Travel	RDT	178	362	0.30	897	0.779
	Control	81	270	0.22	440	
Total	RDT	122	1247	1.02	2,021	0.033
	Control	56	1630	1.33	1,826	

* TSH = Tanzanian shilling; HF = health facility; RDT = rapid diagnostic test. Totals are different than sum of means because of varying sample sizes for each group

† By Kruskal-Wallis test.

**Table 4:** Results of non-parametric bootstrap for confidence interval estimation of patient expenditures, Tanzania*

Type of expenditure	Facility	Mean cost per patient	95% Bias corrected confidence interval
		2008 U. S. dollars	TSH
Expenditure for care pre-HF	RDT	0.07	0.02–0.18
	Control	0.04	0.01–0.12
Drug expenditure at HF	RDT	0.38	0.27–0.53
	Control	0.74	0.52–1.03
Out-patient charges	RDT	0.06	0.05–0.08
	Control	0.08	0.06–0.12
Laboratory fee at HF	RDT	0.20	0.15–0.27
	Control	0.21	0.13–0.32
Post visit expenditure	RDT	0.16	0.04–0.34
	Control	0.06	0.00–0.22
Travel expenditure	RDT	0.30	0.21–0.43
	Control	0.22	0.15–0.31
Total expenditure	RDT	1.02	0.76–1.36
	Control	1.33	0.99–1.77

* TSH = Tanzanian shilling; HF = health facility; RDT = rapid diagnostic test. Totals are different than sum of means because of varying sample sizes for each group

**Table 5:** Costs included in provider perspective analysis, Tanzania

Recurrent costs
Clinical staff salaries
Laboratory technician salaries
Support staff salaries
Consumables
Drug costs
Diagnostics
Electricity
Water
Communication
Capital costs
Building and furnishings
Microscopes
Other equipment

**Table 6:** Provider costs per patient, Tanzania*

Type of cost per patient	Facility	No.	Arithmetic mean		Standard deviation	*P*
			TSH	U. S. dollars	TSH	
Drug cost	RDT	178	1567	1.28	1799	0.014†
	Control	81	2095	1.71	1938	
Facility cost	RDT	4	1926	1.58	903	0.161‡
	Control	2	720	0.59	424	
Total cost	RDT	178	4440	3.63	2019	< 0.001†
	Control	81	2833	2.32	1978	

* TSH = Tanzanian shilling; RDT = rapid diagnostic test. Totals include drug costs, facility level costs, and other patient-specific marginal costs.

† By Kruskal-Wallis test.

‡ By *t*-test. Total includes other marginal costs to the facility.

**Table 7:** Results of non-parametric bootstrap for confidence interval estimation of provider economic costs, Tanzania*

Type of cost (per patient)	Facility	All ages		< 5 Years of age		≥ 5 Years of age	
		Mean cost	95% Bias corrected CI	Mean cost	95% Bias corrected CI	Mean cost	95% Bias corrected CI
Drug cost	RDT	1.28	1.07–1.50	1.19	0.90–1.53	1.38	1.10–1.68
	Control	1.71	1.40–2.08	1.08	0.74–1.57	2.29	1.82–2.79
Total cost	RDT	3.63	3.40–3.89	3.59	3.11–3.95	3.69	3.36–4.03
	Control	2.32	1.99–2.69	1.72	1.37–2.22	2.87	2.39–3.38

* CI = confidence interval. Cost values in are 2008 U.S. dollars.
